# *Toxoplasma gondii* Infection Induces High Mobility Group Box 1 Released from Mouse Macrophages

**DOI:** 10.3389/fmicb.2017.00658

**Published:** 2017-04-24

**Authors:** Hui Wang, Muzi Li, Jing Liu, Jianhai Xu, Qian Han, Qun Liu

**Affiliations:** ^1^National Animal Protozoa Laboratory, College of Veterinary Medicine, China Agricultural UniversityBeijing, China; ^2^Department of Pathogenic Biology, Chengdu Medical CollegeChengdu, China; ^3^Laboratory of Tropical Veterinary Medicine and Vector Biology, Hainan Key Laboratory of Sustainable Utilization of Tropical Bioresources, Hainan UniversityHaikou, China

**Keywords:** *Toxoplasma gondii*, HMGB1, IFN-γ, proinflammatory response, caspase-1, inflammasome

## Abstract

High mobility group box 1 (HMGB1) is abundantly expressed in intracellular engaged DNA binding ability. However, more importantly, it is a weapon against infection through proinflammatory response and immune regulation while released to extracellular. *Toxoplasma gondii* causes inflammatory pathological changes including ileitis and encephalitis in chronic infection. To investigate whether HMGB1 contributes to the toxoplasmosis lesions, we examined HMGB1 changes during *T. gondii* infection. The results showed that HMGB1 transcription was down-regulated in the murine macrophage ANA1 cell line and mouse peritoneal macrophages (PMΦs) after *T. gondii* inoculation, but up-regulated in the IFN-γ treated macrophages and the intraperitoneal exudate cells from the *T. gondii* infected mice. The content of intracellular HMGB1 are basically consistent with the transcription levels in ANA1 assay, while there were no obvious changes in the mouse PMΦs. Both ANA1 and mouse PMΦs released HMGB1 after parasites infection, and no obvious HMGB1 aggregation in cytoplasm compare to the IFN-γ treatment group. Furthermore, we demonstrated that *T. gondii* invasion led to HMGB1 release, which was dependent on the Caspase 1 activity. These finding should promote to further investigate the functions of extracellular HMGB1 in the toxoplasmosis.

## Introduction

*Toxoplasma gondii* is an obligate intracellular protozoan, which invades almost all nucleated cells and resists phagosome-lysosome fusion killing through forming parasitophorous vacuoles (PV). Toxoplasmosis is an opportunistic disease in various animals and humans (Dubey, 1996). European and North American strains of *T. gondii* belong to three distinct clonal lineages: types I, II, and III, which differ genetically by 1% or less (Sibley and Ajioka, 2008). In laboratory mice, type I (high virulence) strains are categorically lethal with an LD100 = 1, whereas the LD50 of type II (intermediate virulence) and III strains (low virulence) are ∼103 and 105, respectively (Sibley and Boothroyd, 1992; Sibley and Ajioka, 2008). Many genes have been identified to be responsible for the difference between biological phenotypes, virulence and dissemination (Dubremetz and Lebrun, 2012). *T. gondii* can carefully regulate immune responses and host cell effector mechanisms, mostly by its ROPs (ROP18, ROP5, ROP17, ROP16, and ROP38), GRAs [GRA7 (Alaganan et al., 2014), GRA15], Profilin, Cyclophilin-18 (reviewed in Melo et al., 2011; Pifer and Yarovinsky, 2011), after establishing homeostasis, and even life-long chronic infection. GRA15type II directly activates NF-κB in the infected cells; ROP16type II transiently activates STAT3/6, and the non-virulent form of ROP18 and ROP5 complex does not effectively block the recruitment of IRGs to the PV. So type II *T. gondii* is very effective in producing massive pro-inflammatory cytokines early after infection and inducing a Th1 type polarization response (Denkers and Gazzinelli, 1998; Melo et al., 2011). As the most common type of isolates from all over the world, type II *T. gondii* infection leads to controlled intracellular parasite growth, but susceptible animals die of acute severe ileitis (Liesenfeld et al., 1996) and encephalitis in the infection.

The inflammasome has recently gained attention, as defects in this pathway are associated with uncontrolled *T. gondii* growth (Lees et al., 2010; Witola et al., 2011). Further studies showed that single nucleotide polymorphisms (SNPs) of human NLRP-1 are associated with susceptibility to congenital Toxoplasmosis (Witola et al., 2011). Another possible receptor involved in activation of the inflammasome following *T. gondii* infection is the P2X7 (Jamieson et al., 2010), a purinergic receptor, which after binding to ATP and subsequent efflux of intracellular K+ leads to activation of the inflammasome (Stutz et al., 2009; Schroder and Tschopp, 2010). Activation of inflammasome have different roles in distinct animals and humans, it seems to be deleterious in the mouse model (Hitziger et al., 2005), but may help to eliminate the parasite in rats and humans (Cavailles et al., 2006; Correa et al., 2010; Lees et al., 2010). The delicate balance between pro- and anti-inflammatory signals is necessary to guarantee survival of both the host and parasite.

NLRP-1 is the sensor of *T. gondii* infection, which induces CASP1-dependent response in mice (Ewald et al., 2014), but the mechanism is unknown. Particularly, it needs to be clarified whether *T. gondii* secretes a protease, which has a cleavage site specificity distinct from anthrax lethal toxin (LeTx), into the cytosol of infected cells, where it would cleave and activate NLRP-1. Inflammasome stimulation activates caspase-1, which cleaves the proforms of IL-1β and IL-18 generating bioactive cytokines that subsequently play the pro-inflammatory functions (Guarda and So, 2010; Rathinam et al., 2012). Actually, IL-1 family members are not the unique group of pro-inflammatory proteins. Following the formation and activation of inflammasomes, some danger-associated molecular patterns (DAMPs) are also released from cells, which lack classical secretion signals and even though they may not strictly be classified as cytokines. However, different downstream consequences in inflammation and immunity are endowed while they were secreted extracellularly (Keyel, 2014). As the best-studied DAMPs, High Mobility Group Box 1 (HMGB1) is an abundant non-histone nuclear protein, which has a minor-groove binding enhancer involved to cell development and differentiation. Upon release from the cell, HMGB1 binding to multiple PRRs can lead to inflammation and immunity regulation or many other biological functions (Lotze et al., 2007).

Contrast to typical cytokines, DAMPs bind with a lower affinity and lack dedicated receptors (Carta et al., 2013). Furthermore, HMGB1 secretion from LPS-primed macrophages requires the inflammasome components apoptotic speck protein, which contains a caspase activation and recruitment domain (ASC), caspase 1 and Nalp3, whereas HMGB1 secretion from macrophages infected *in vitro* with *Salmonella typhimurium* was dependent on caspase-1 and Ipaf-1. Nevertheless, HMGB1 is not a direct substrate of caspase-1 (Lamkanfi et al., 2010). Recently, a new study indicated that HMGB1 is sensitive to processing by caspase-1. Caspase-1-mediated HMGB1 cleavage (resulting in production of the A-box fragment) reversed apoptosis-induced tolerance through binding to the receptor for advanced glycation end products (RAGE) in DCs. HMGB1 can be specifically processed by caspase-1, but not other caspases (-2, -3, -5, -7, -9, or -11) (LeBlanc et al., 2014), nevertheless, caspase 1 itself is confusing regarding *Toxoplasma* infection. These findings raised an interesting question, that whether HMGB1 involved to Toxoplasmosis pathogenesis, and the purpose of this study was to verify can the sensing of *T. gondii* infection lead to the release of HMGB1? and if so, is this release dependent on caspase-1 processing?

## Materials and Methods

### Ethics Statement

All experiments with animals in this study were performed in accordance with the recommendations in the Guide for the Care and Use of Laboratory Animals published by the Ministry of Science and Technology of China. Animal ethics in all experimental procedures were approved by the Institutional Animal Care and Use Committee of China Agricultural University. All efforts were made to minimize animal suffering.

### Parasites and Cell Culture

Murine macrophage ANA1cells (Cox et al., 1989) were cultured in complete DMEM, as described previously (Soloviev et al., 2002). ANA-1 cells were produced from bone marrow macrophages of the C57BW6 mice by infection with the J2 recombinant retrovirus expressing v-myc/v-raf. The cells express markers of the differentiated macrophage, including Ly-5, Mac-I, and FcyR, but do not express either B or T lymphocyte cell markers. *T. gondii* of Pru strain was maintained *in vitro* by serial passage on confluent HFF monolayers in DMEM containing 25 mM glucose and 4 mM glutamine supplemented with 10% newborn calf serum (NCS, Hyclone, USA) (Wang et al., 2014). The cells were incubated at 37°C with 5% CO_2_ in a humidified incubator. The medium was changed 6 h after inoculation, and thereafter every 24 h.

### Mice Infection

Inbred strains of 6- to 8-week-old Balb/c female mice (center of Beijing experimental animals) were used for Pru strain *T. gondii* infection. Briefly, Pru strain tachyzoites were harvested and purified using a 5 μm filter, and infected at 2 × 10^6^/mouse dose through intraperitoneal injection. 5 mL PBS was injected into the abdominal cavity for acquisition of mouse peritoneal exudate cells and exudate at 6, 12, 24, 48, 72, and 96 h after infection, lavage fluid were centrifuged at 600 *g* for 10 min at room temperature, peritoneal exudate cells in the pellet and supernatant were collected, respectively. Supernatants were then precipitated and concentrated by the precipitation reagent (equal volumes of acidified acetone and methanol) for HMGB1 assay. Cells were harvested for the examination of transcription and expression of HMGB1.

### Isolation of PMΦs and *T. gondii* Inoculation

The mice PMΦs were isolated as described previously (Chakraborty et al., 2013). Pru strain tachyzoites were harvested and purified using a 5 μm filter. The parasites were inoculated on to the macrophage monolayers at a parasite to host cell ratio of 5:1. Supernatants were collected at the indicated time points, and then precipitated and concentrated as described above. Cells were harvested for the examination of transcription and expression of HMGB1.

### Activation and Inhibitor Studies

Mouse PMΦs and ANA1 monolayers were cultured in complete RPMI 1640 medium in 24 well culture plates (1 × 106 cells/well) with 10 ng/mL IFN-γ (Sigma–Aldrich, USA) or 2 μM Z-VAD-FMK (Bioversion, USA) or ac-YVAD-cmk (Sigma–Aldrich, USA) for 6 h. All chemicals were used at non-cytoxic concentrations, which means macrophages with >90% viability were examined by Trypan blue staining after treatment. Thereafter, the medium was refreshed and *T. gondii* tachyzoites were inoculated at a parasite to host cell ratio of 5:1. Further incubation was carried out for different time periods as indicated. The supernatant and cell pellet were collected separately for HMGB1 assay as described above.

### Real-Time PCR

Total RNA was isolated from ANA1 cells and mouse PMΦs that were uninfected or infected by pru parasites using the RNA isolation kit version 2.11 (Takara Biotechnology, Dalian, Co., Ltd) according to the manufacturer’s directions, and treated with DNase I (Takara) to remove any residual genomic DNA. cDNAs were synthesized using Oligo (dT)18 and random 6-mers using the cDNA Synthesis Kit (Takara). Specific primers were designed using Primer Premier 5.0, including primers for Mus musculus HMGB1 (GI: 114326547, forward: 5′-GAGACCAAAAAGAAGTTCAAGGAC-3′ and reverse: 5′-TGCAACATCACCAAT GGATAA G-3′), and GAPDH (forward: 5′-GGTGAAGGTCGGTGTGAACG-3′ and reverse: 5′-CTCGCTCCTGGAAGATGGTG-3′). Specificity of these primers was evaluated using conventional real-time (RT)-PCR. Quantitative RT-PCR (ΔΔCt method) was performed using the ABI Prism 7500 System (Applied Biosystems Inc., USA) with Tli RNaseH Plus kit (Takara). The RNA concentration was normalized to GAPDH.

### Immunofluorescence Assays

Immunofluorescence assays (IFA) for HMGB1 localization and translocation in ANA1 and mouse PMΦs were performed as described previously (Wang et al., 2014). Briefly, cells cultured on coverslips and subjected to different treatments were fixed, permeabilized, and blocked prior to incubation with the rabbit anti-HMGB1 monoclonal antibody (ABcam, UK, diluted 1:200 in 1% BSA-PBS) for 1 hr at 37°C. The coverslips were washed and then incubated for 1 h at 37°C with FITC-conjugated goat anti-rabbit IgG (H+L) diluted to 1:50 in PBS with 1% BSA (Proteintech, USA). Nuclei were stained with Hoechst 33258 (Sigma–Aldrich, USA) and coverslips were subsequently mounted onto slides. Fluorescence images were obtained through the Leica microsystem (Leica TCS SP5 II, Germany) and epifluorescence optics were obtained using an oil immersion lens with 63× magnification. Collected images and MIFs (mean index of fluorescence) were processed and analyzed using the LAS AF lite 2.2.0 software (Leica). Mouse anti-TgHMGB1a antibodies were used to trace the *T. gondii* (Wang et al., 2014).

### Western Blotting of HMGB1

To examine HMGB1 protein, concentrated supernatant and cells (*T. gondii* infected ANA1 cells and mouse PMΦs), peritoneal lavage cells and fluid from the *T. gondii* infection mice were lysed using RIPA buffer (Beyotime, Beijing) with a cocktail of protease inhibitors. The lysate was loaded on 12% (w/v) SDS-PAGE. After electrophoresis, separated strips were transferred onto polyvinylidene fluoride (PVDF) membranes (Millipore, USA) together with a visible prestained protein marker. Membranes were blocked overnight with 5% (w/v) skim milk in PBS at 4°C, and then incubated with the rabbit anti-HMGB1 monoclonal antibody (diluted 1:600 by 3% BSA-PBS). After washing in PBST (0.5‰ Tween-20) for three times, the membranes were incubated for 1 h with goat anti-rabbit IgG (H+L) horseradish peroxidase (HRP)-labeled secondary antibody (Proteintech, USA) diluted to 1:10,000 in 3% BSA-PBS. Finally, the blot was developed using enhanced chemiluminescence reagents (Co Win Biotech Co., Ltd., Beijing). Antibodies against GAPDH were used as endogenous control.

### Statistical Analysis

Statistical test were performed in Statistical Product and Service Solution 22.0 (SPSS 22.0, IBMCo., USA). Two-tailed *P*-value was calculated, and *P* < 0.05 was consider significant.

## Results

### *T. gondii* Infection Induces Downregulation of HMGB1 Transcription in ANA1 Cells, Where Asthe Transcription of HMGB1 Was Upregulated in Peritoneal Exudates Cells at the Early Stage of *T. gondii* Infected Mice

**FIGURE 1:**
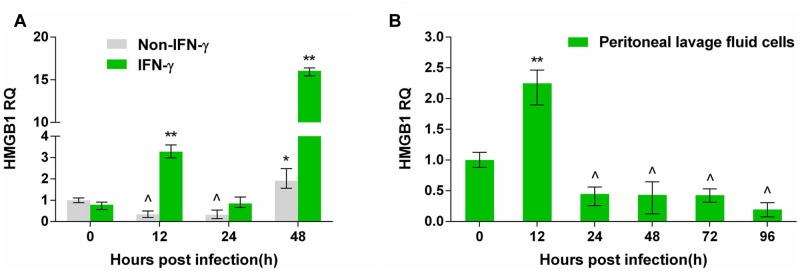
**The transcription changes of high mobility group box 1 (HMGB1) by quantitative-PCR in Pru *Toxoplasma gondii* infected ANA1 cells and peritoneal exudate cells in *T. gondii*-infected mice.** **(A)** HMGB1 transcription in Pru infected normal and IFN-γ primed ANA1 cells. **(B)** HMGB1 transcription in the peritoneal exudate cells from Pru infected mice. Comparative statistics performed between 0 h and the other time point, ^∗^indicates rose compared to 0 h, ^∗^*P* < 0.05, ^∗∗^*P* < 0.01; ˆ indicates decreased compared to 0 h, *P* < 0.05.

Pru strain tachyzoites were inoculated into ANA1 cells with or without IFN-γ pretreatment. HMGB1 transcription continuously declined in ANA1 cells over the first 24 h after *T. gondii* infection, which may suggest inhibition of cell growth by *T. gondii* invasion. HMGB1 transcription rose at 48 h (**Figure 1A**), possibly related to cell death in the late stage of *T. gondii* infection *in vitro*. In parallel tests, HMGB1 transcription increased at 12 h after *T. gondii* infection, and then declined before a sharp increase at 48 h in the IFN-γ pretreated ANA1 cells (**Figure 1A**). To verify whether the changes occurred *in vivo*, peritoneal exudate cells from the *T. gondii* infected mice were gathered for examination. Quantitative-RT-PCR results indicated that, in the early 12 h after infection, HMGB1 transcription began to rise, which is similar to the test *in vitro* using IFN-γ treated ANA1 cells (**Figure 1A**), but then decreased gradually to below the basal level, and there were no upward trends again in the detecting period (**Figure 1B**). These results probably implied that IFN-γ lead to upregulated HMGB1 transcription at the early stage of pru infection *in vivo*.

### *T. gondii* Infection Induces HMGB1 Secretion Both *In Vitro* and *Vivo*

**FIGURE 2:**
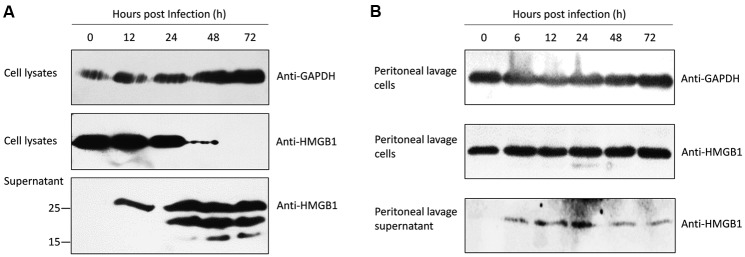
**Western blots of the intracellular and secreted HMGB1 protein after Pru *T. gondii* infection.** **(A)** Intracellular HMGB1 and secretion examination after Pru infected ANA1 cells with the anti-HMGB1 monoclone antibody at the indicated time points. **(B)** Western blot assay in the intracellular HMGB1 in the peritoneal lavage cells and HMGB1 secretion in the fluid from Pru infected Balb/c mice at the indicated time points. Anti-GAPDH monoclone antibody was used as an endogenous control.

To investigate whether *T. gondii* infection induces HMGB1 secretion, supernatant and the infected ANA1 cells were collected for western blot. At the early stage (∼24 h), consistent with the transcription level of HMGB1 (**Figure 1A**), intracellular HMGB1 gradually decreased after Pru infected the ANA1 cells (**Figure 2A**). Even at 48 h, the specific HMGB1 strip almost disappeared, but the transcription level of HMGB1 was dramatically rising at 48 h (**Figure 1A**). Simultaneously, Ana1 cells began to secret HMGB1 to the supernatant soon after infection (**Figure 2A**). Interestingly, there was only one specific band about 25 kD of HMGB1 in the supernatant at 12 h as expected, but afterward two smaller molecular weight bands emerged, with a trend of increasing with time. Based on the high specificity of Anti-HMGB1 monoclonal abs (**Supplementary Figure S1**), we proposed that the two new bands might be formed by the splicing of HMGB1 by caspase (LeBlanc et al., 2014) or unknown protein kinases, or other shear degradation mechanisms. Our finding showed that *T. gondii* infection induces HMGB1 secretion in ANA1 cells; however, the function of HMGB1 release and processing in *T. gondii* infection need further investigations.

To validate the putative changes of HMGB1 during *T. gondii* infection *in vivo*, peritoneal exudate cells of the pru strain tachyzoites infected mice were collected for examination. No significant changes of cellular HMGB1 were observed in the total peritoneal exudate cells lysates during *T. gondii* infection (**Figure 2B**). Further peritoneal lavage fluid results showed that HMGB1 was secreted at 6 h after infection and continuous secretion was detected in the following periods (**Figure 2B**), similarly to the *in vitro* assay using ANA1 cells (**Figure 2A**). Although the amounts were less, which possibly as a result of HMGB1 binding to its receptors *in vivo* (detected in the cell lysates).

### *T. gondii* Infection Induces Intracellular HMGB1 Changes and Releases in Mouse PMΦs

**FIGURE 3:**
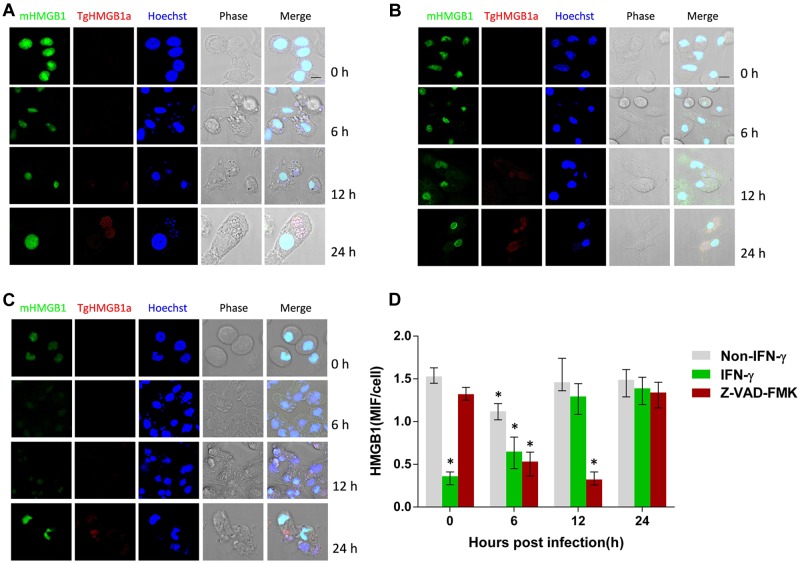
**High mobility group box 1 distribution in the *T. gondii* infected mouse PMΦs with different treatments.** **(A)** IFA examination of the HMGB1 distribution in the *T. gondii* infected normal mouse PMΦs, **(B)** mouse PMΦs with IFN-γ and **(C)** caspase inhibitor treatment, Murine HMGB1 was labeled with anti-mouse HMGB1 monoclonal antibody and the *T. gondii* HMGB1 (TgHMGB1a) was labeled with anti-TgHMGB1a 4E sera. Scale bar is 10 μm. **(D)** MIF (mean intensity of fluorescence) statistical analysis of the Pru infected peritoneal lavage macrophages of mice with different treatments; HMGB1 were labeled through IFA. Comparative statistic performed between 0 h and the other time point, ^∗^*P* < 0.05.

**FIGURE 4:**
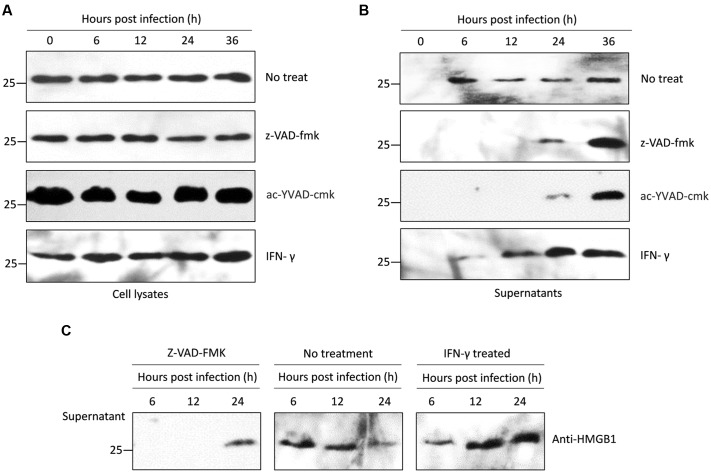
**Western blots of intracellular and secreted HMGB1 in mouse PMΦs infected with *T. gondii in vitro*.** **(A)** Intracellular HMGB1 were examined in the Pru infected mice PMΦs with a different treatment-through western blot using the anti-HMGB1 monoclone antibody. Anti-GAPDH monoclone antibody was used as control. **(B)** Western blot assay in the secreted HMGB1 in the Pru infected mice PMΦs with different treatment. **(C)** HMGB1 secretion were examined after RH infected mice PMΦs.

To detect possible phenotype changes of ANA1 cell line and to further investigate the cell source of HMGB1 secretion in the peritoneal cavity of *T. gondii* infected mice, mouse PMΦs were isolated for *T. gondii* infection. Results of IFA and MIFs statistics showed that the intracellular HMGB1 of IFN-γ treated PMΦs was at least reduced by half compared with normal PMΦs (**Figure 3**), while *T. gondii* infection induced the intracellular HMGB1 increasing gradually again. In parallel experiments, HMGB1 in untreated PMΦs slightly decreased at 6 h post *T. gondii* infection, and then rose to the basal level at 12 h (**Figure 3A**). We also monitored HMGB1 in supernatant and HMGB1 released after *T. gondii* infection from 6 to 36 h (up panel in **Figure 4B**). Western blot elicited only one HMGB1 specific band with the expected molecular weight (about 25 kD), differing from in the ANA1 test (**Figure 2A**). These results demonstrated that the macrophage was one source of HMGB1 secretion in *T. gondii* infection, and *T. gondii* infection can possibly resist IFN-γ mediated cell killing, which leads to HMGB1 expression recovery in the ANA1 cells and PMΦs.

### Active Caspase-1 Is Required for *T. gondii* Induced HMGB1 Release

We explored whether *T. gondii*-induced HMGB1 release is dependent on NLRP1/3-Caspase-1 inflammasomes pathways. Primary mouse PMΦs were pretreated with a cell permeable irreversible pan-caspase inhibitor Z-VAD-FMK and or ac-YVAD-cmk, which potentially inhibits caspase-1 activity (Dostert et al., 2009; Chakraborty et al., 2013). Intracellular HMGB1 had no obvious changes in caspase inhibited mouse PMΦs without inoculation, while after parasitic infection, the nuclear HMGB1 initially decreased (**Figures 3C,D**). Unlike the IFA visualization and MIFs data, western blot showed that the total HMGB1 of mouse PMΦs with different treatment were not significantly different after *T. gondii* infection (**Figure 4A**). The result of the IFA and MIF statistics should accurately demonstrate that nuclear HMGB1 decreased and dispersion after one cell was infected by *T. gondii* parasites, while the western blot only shows the total cell HMGB1, and cells without invasion in the experiment can’t be excluded.

We further detected HMGB1 in the supernatant of mouse PMΦs culture with IFN-γ and z-VAD-fmk or ac-YVAD-cmk. After pru strain parasites infection stimulated a sustainable time-independent release of HMGB1 from the normal PMΦs (up panel of **Figure 4B**), and as expected, a large number of HMGB1 were released upon *T. gondii* infected the IFN-γ primed PMΦs (bottom panel of **Figure 4B**), both the z-VAD-fmk or ac-YVAD-cmk inhibitor treatment delayed and suppressed HMGB1 release. HMGB1 was detectable in the supernatant only after 24 h (middle panel of **Figure 4B**) possibly because of the egress of parasites which probably damage cell membrane. To verify whether this phenomenon is *T. gondii* strain-specific, RH strain tachyzoites was used as infecting parasites, and similar results were obtained (**Figure 4C**). All these results indicated that *T. gondii*, independent of strains, induces HMGB1 release in macrophages, and the release is probably dependent on the Caspase1-inflammasome pathway.

## Discussion

*Toxoplasma gondii* infection has been shown to activate NLRP3/1-infammasomes both in rat and mouse (Ewald et al., 2014; Gorfu et al., 2014). Human susceptibility to congenital toxoplasmosis has also been proved to link to SNPs of NLRP-1; however, the mechanism by which *T. gondii* activates NLRP3/1-infammasomes is poorly understood. Inflammasome stimulation further activates caspase-1, which cleaves the proforms of IL-1β and IL-18 to generate bioactive cytokines. *T. gondii* is known to induce IL-1β and IL-18 secretion, both of which serve to amplify IFN-γ production by NK cells (Hunter et al., 1995; Cai et al., 2000). In this study, we checked the TNF-α, IL-1β, and IL-18 at the transcription level; however, there were no significant difference after pru strain parasites infection (data not shown), which probably indicated that the secretion of IL-1β and IL-18 probably came from the stored precursor in the cytoplasm. But whether the parasite directly activates the inflammasome or modulate caspase-1 activity remains to be elucidated.

Mammalian cells export most proteins by the endoplasmic reticulum/Golgi-dependent pathway; however, some leaderless proteins are secreted via unconventional mechanisms. As described above, caspase-1 has a well-established function as a general regulator of stress induced unconventional secretion for a number of cytokines such as proinflammatory factors IL-1β, IL-18, and IL-33 (Dinarello, 2002; Ogura et al., 2006). Caspase-1 itself is activated by inflammasomes. The leaderless proteins are not substrates of the protease, but actually can be secreted through their physical interactions, and many of these unconventional secreted proteins are involved in inflammation, cytoprotection, or tissue repair (Keller et al., 2008). In this study, we have observed that *T. gondii* infection is able to induce HMGB1 release in mouse PMΦs, and pharmacological inhibitors for caspase-1 abrogated this process (**Figure 4B**). This effect was likely mediated via physical interaction between HMGB1 and active caspase-1 (p10 and p20), which can act as a carrier for HMGB1 release, as demonstrated by Chakraborty et al. (2013). Thus, we proposed that *T. gondii* infection induces HMGB1 release from mouse PMΦs which is likely mediated by NLRP1/3-caspase-1-inflammasome activation. However, we also speculated an alternative possibility that cells can sense *T. gondii* invasion through NLRP-1, and then activate caspase-1. Caspase-1 coupling inflammasomes mediate inflammatory reaction, which leads to secrete proinflammatory cytokines including TNF-α, IL-1β, IL-12, and IFN-γ. These cytokines finally promote the dangerous signal, “alarmins” molecular HMGB1 release probably at the later stages.

The reason for transcriptional upregulation at the early stage of *T. gondii* infection could be due to the fact that HMGB1 *per se* is well established to interact with the minor groove of DNA and enables physical interactions between DNA and a variety of molecules, including p53, NF-κB and steroid hormone receptors (Campana et al., 2008). Activation of these genes by *T. gondii* infection directly induces macrophages to upregulate the transcription of HMGB1, which may be related to the activation of macrophages. The decrease of HMGB1 possibly indicates inhibition of cell growth.

HMGB1 is a ubiquitous protein located inside and outside of cells that play critical roles in various physiological and pathological processes including cell development, differentiation, inflammation, immunity, metastasis, metabolism, autophagy, and death. As far as we know, HMGB1 plays a critical role in the inflammation related diseases. The inhibition or neutralization of HMGB1 have been a new therapeutic target for the inflammatory diseases. For the multifunction of HMGB1, *T. gondii* infection induced release of HMGB1 may be a protective mechanism of the body to resist the pathogen, but also a possible promoter of serious pathological responses. As shown in the mouse infection model, strong inflammation responses are arising at the inoculation foci, and it can be presumed that the proinflammatory reaction may be related to the high expression and secretion of HMGB1 at the early stage of infection. Therefore, the delicate mechanism and physiological functions of HMGB1 release in *T. gondii* infection should be further investigated.

## Conclusion

The mouse PMΦs released HMGB1 after *T. gondii* infection, which is partially dependent on the NLRP-Caspase 1-inflammasome pathway. However, the functions of extracellular HMGB1 need to be further investigated in the toxoplasmosis, maybe especially in the encephalitis.

## Author Contributions

Conceived and designed the experiments: HW, QL, and JL. Performed the experiments: HW, ML, and JX. Analyzed the data: HW, JL, QH, and QL. Contributed reagents/materials/analysis tools: QL. Wrote the paper: HW and QL.

## Conflict of Interest Statement

The authors declare that the research was conducted in the absence of any commercial or financial relationships that could be construed as a potential conflict of interest.

## Supplementary Material

**FIGURE S1 | Examine the reactivity of the anti-HMGB1 monoclonal antibody through IFA and western blot.** **(A)** HMGB1 distribution in HFF, **(B)** macrophages from mice peritoneal lavage and **(C)** ANA1 cells. IFA using the anti-HMGB1 monoclonal antibody showed that HMGB1 concentrated in nucleus of HFF, macrophages from mice peritoneal lavage and ana1 cells. The bar is10 μm. **(D)** Western blot assay the HMGB1 in different cell types, using the anti-HMGB1 monoclonal antibody can recognize ∼25 kD protein (HMGB1) in the macrophages from mice peritoneal lavage, ana1 cells and HFF lysetes, respectively, but not in *T. gondii* total antigens.
