# Supplementary figures and images for: Toxoplasma gondii Infection Induces High Mobility Group Box 1 Released from Mouse Macrophages
Source: Front Microbiol. 2017 Apr 24;8:658. doi: 10.3389/fmicb.2017.00658 (PMC5402041; doi:10.3389/fmicb.2017.00658)

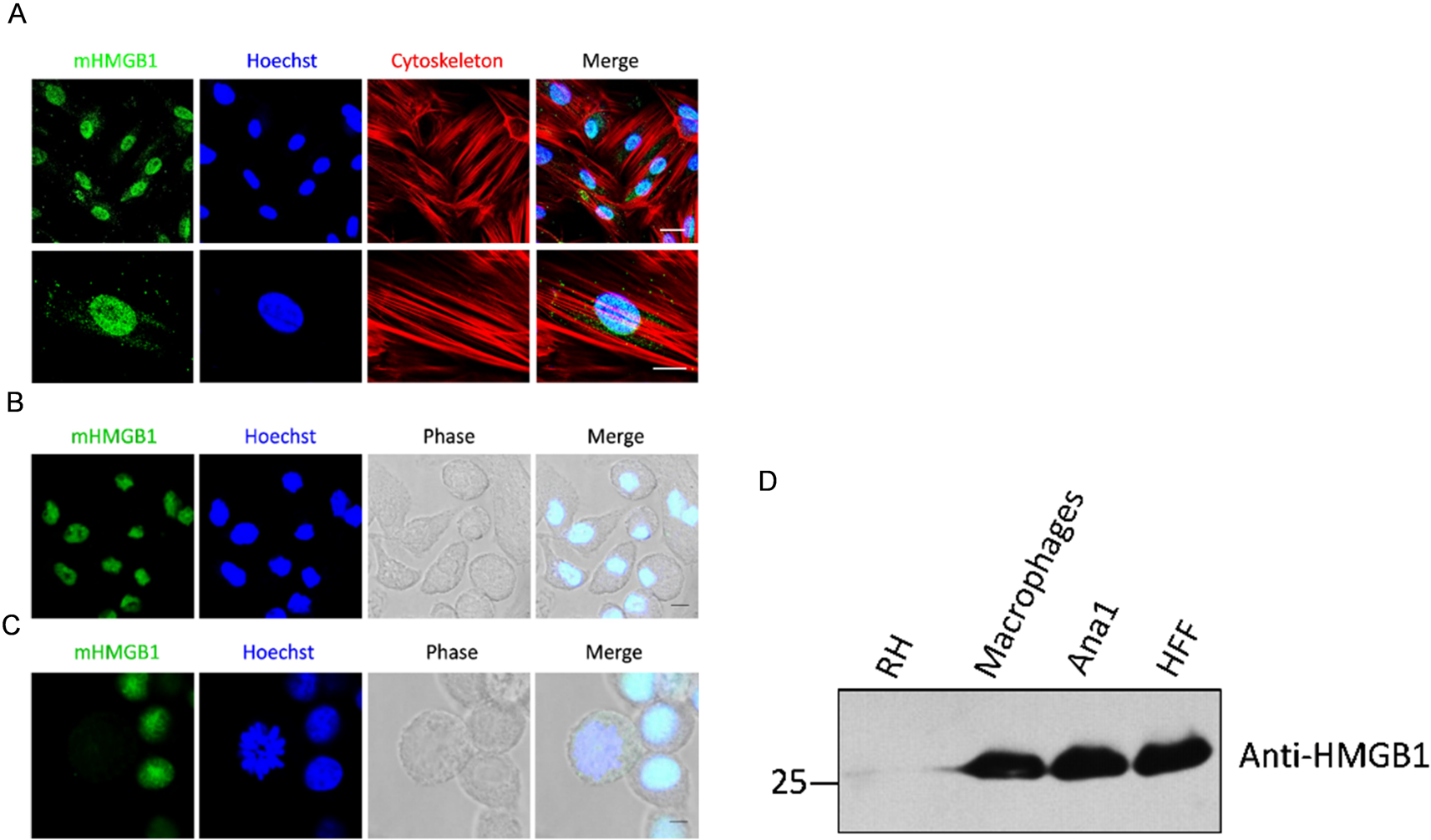

Supplement: Supplementary file 1 [file Image_1.TIF]
